# Phosphatidylinositol 4,5-bisphosphate (PIP_2_) facilitates norepinephrine transporter dimerization and modulates substrate efflux

**DOI:** 10.1038/s42003-022-04210-1

**Published:** 2022-11-17

**Authors:** Dino Luethi, Julian Maier, Deborah Rudin, Dániel Szöllősi, Thomas J. F. Angenoorth, Stevan Stankovic, Matthias Schittmayer, Isabella Burger, Jae-Won Yang, Kathrin Jaentsch, Marion Holy, Anand Kant Das, Mario Brameshuber, Gisela Andrea Camacho-Hernandez, Andrea Casiraghi, Amy Hauck Newman, Oliver Kudlacek, Ruth Birner-Gruenberger, Thomas Stockner, Gerhard J. Schütz, Harald H. Sitte

**Affiliations:** 1 Institute of Pharmacology, Center for Physiology and Pharmacology, Medical University of Vienna, Waehringer Strasse 13A, 1090 Vienna, Austria; 2 Institute of Applied Physics, TU Wien, Lehargasse 6, 1060 Vienna, Austria; 3 Institute of Chemical Technologies and Analytics, TU Wien, Getreidemarkt 9, 1060 Vienna, Austria; 4 Physics Program, New York University Abu Dhabi, Saadiyat Island, 129188 Abu Dhabi, United Arab Emirates; 5 Medicinal Chemistry Section, Molecular Targets and Medications Discovery Branch, National Institute on Drug Abuse – Intramural Research Program, Baltimore, MD 21224 USA; 6 Department of Pharmaceutical Sciences, University of Milan, Via Luigi Mangiagalli 25, 20133 Milan, Italy; 7 Diagnostic and Research Institute of Pathology, Medical University of Graz, Neue Stiftingtalstrasse 6, 8010 Graz, Austria

**Keywords:** Single-molecule biophysics, Transporters in the nervous system, Total internal reflection microscopy

## Abstract

The plasmalemmal norepinephrine transporter (NET) regulates cardiovascular sympathetic activity by clearing extracellular norepinephrine in the synaptic cleft. Here, we investigate the subunit stoichiometry and function of NET using single-molecule fluorescence microscopy and flux assays. In particular, we show the effect of phosphatidylinositol 4,5-bisphosphate (PIP_2_) on NET oligomerization and efflux. NET forms monomers (~60%) and dimers (~40%) at the plasma membrane. PIP_2_ depletion results in a decrease in the average oligomeric state and decreases NET-mediated substrate efflux while not affecting substrate uptake. Mutation of the putative PIP_2_ binding residues R121, K334, and R440 to alanines does not affect NET dimerization but results in decreased substrate efflux that is not altered upon PIP_2_ depletion; this indicates that PIP_2_ interactions with these residues affect NET-mediated efflux. A dysregulation of norepinephrine and PIP_2_ signaling have both been implicated in neuropsychiatric and cardiovascular diseases. This study provides evidence that PIP_2_ directly regulates NET organization and function.

## Abstract (web summary)

Phosphatidylinositol 4,5-bisphosphate (PIP_2_) directly regulates norepinephrine transporter (NET) dimer stabilization at the plasma membrane. Furthermore, PIP_2_ interaction with NET affects efflux.

## Introduction

The human norepinephrine transporter (hNET) regulates noradrenergic signal transduction by high-affinity uptake of norepinephrine from the synaptic cleft. NET belongs to the sodium- and chloride-dependent solute carrier (SLC) 6 family transporters (SLC6A2 gene^1^), which also include the monoamine transporters for dopamine (DAT; SLC6A3) and serotonin (SERT; SLC6A4)^2^. In addition to its expression in the central nervous system, NET is expressed in the peripheral nervous system, adrenal glands, and the placenta^2,3^. NET has opposing effects on cardiovascular sympathetic regulation in the brain and in the periphery and modulates the distribution of sympathetic activity between vasculature, heart, and kidney^4^. Moreover, NET is an important target of a variety of prescription drugs^2^ and drugs of abuse^5^. Impaired cardiac NET function has been associated with several cardiac health conditions, such as congestive heart failure^4^. Single nucleotide polymorphisms in the NET gene have been linked to orthostatic intolerance and major depression^6–8^. Even though depression is considered a heterogenous disease, various symptoms are specifically attributed to the norepinephrine system^9,10^. Mental disorders are a large and growing social and economic burden^11^; however, the efficacy of current treatment options is far from optimal, with up to 30% of patients suffering from depression not responding to conventional treatments^12^. The need for a better understanding of disease mechanisms and the development of more efficacious treatments is therefore evident.

Monoamine transporter regulation and function is driven by the interplay of various kinases, proteins, and lipids^13–15^. An imbalance of such biomolecules may result in transporter malfunction and subsequent disease. An important role in monoamine transporter function and quaternary organization has been attributed to the phospholipid phosphatidylinositol 4,5-bisphosphate (PIP_2_)^16–20^. PIP_2_ is present in the intracellular leaflet of the plasma membrane and constitutes around 2% of the inner leaflet lipid composition^19^. Besides its importance in the second messenger pathway, PIP_2_ regulates membrane proteins^21^, including ion channels^22–27^, receptors^28^, and transporters^16,18,20^. Hitherto, however, it remains unknown to which extent PIP_2_ modulates NET function and organization. Interestingly, altered PIP_2_ and norepinephrine signaling have both been associated with several diseases, including neurological diseases such as bipolar disorder or Alzheimer’s disease^29–34^; this manifests the need for further investigation of this lipid-protein connection.

PIP_2_ kinetically traps SERT but not DAT oligomers at the plasma membrane^17,19,35,36^. PIP_2_ depletion or mutation of the PIP_2_ binding sites K352 and K460 to uncharged residues causes equilibration of the different higher-order SERT oligomers^17^. Monoamine transporter oligomerization has been proposed to be a trafficking quality control mechanism^37^ and to be a prerequisite for drug-induced neurotransmitter efflux^38^. Furthermore, DAT oligomerization was suggested to play a role in cocaine tolerance development^39^ and to be involved in clathrin-independent endocytosis and subsequent endosomal retention of DAT in cells exposed to the activated CDC42 kinase 1 (Ack1) inhibitor AIM-100^40^. In DAT, PIP_2_ was demonstrated to interact with the arginine residue R443 on intracellular loop 4, which electrostatically regulates dopamine efflux in coordination with the N-terminus^18,41^. Similarly, the action of substrate-type serotonergic stimulants depends on the binding of PIP_2_ to SERT^16^. Specifically, the cytoplasmic residues K352 on intracellular loop 3, K460 on intracellular loop 4, and to a lesser extent R144 on intracellular loop 1 have been identified to interact with PIP_2_^16^.

**Fig. 1:**
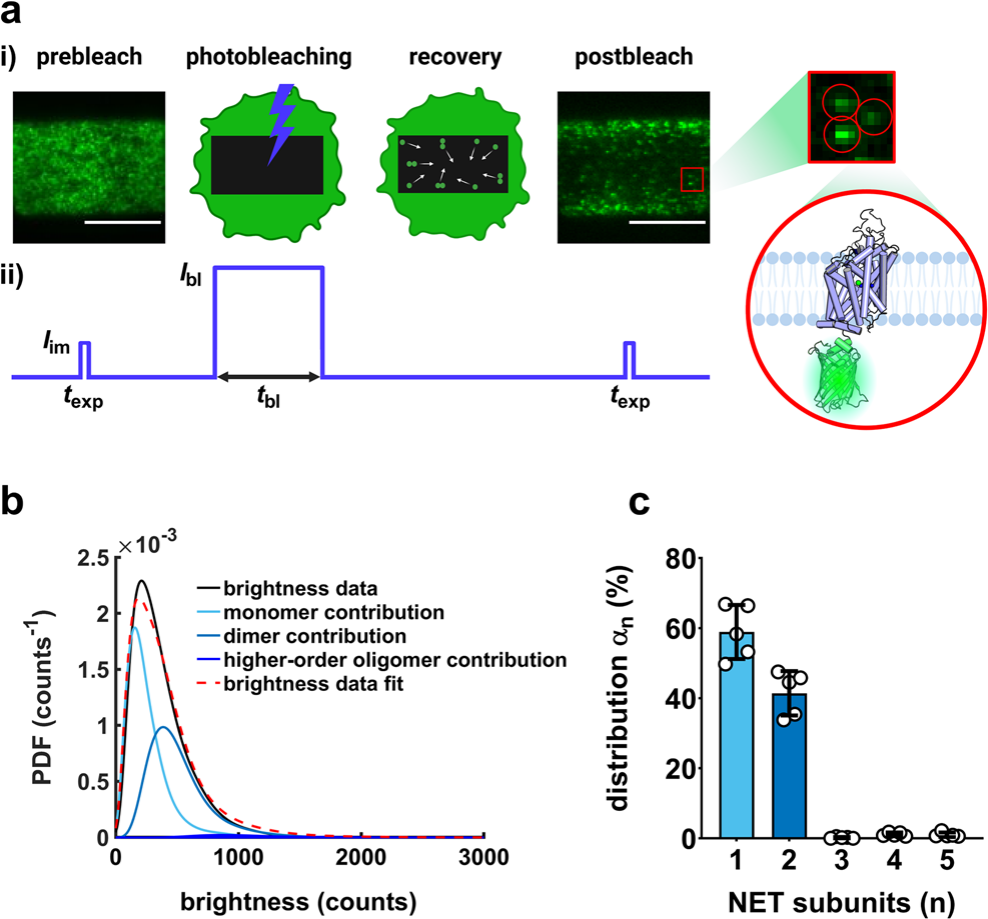
Determination of mGFP–hNET oligomer distribution using single-molecule brightness analysis. **a** Concept of TOCCSL runs in mGFP-hNET-transfected CHO cells. i) A prebleach image is recorded and an aperture-restricted area of the bottom cell membrane is subsequently photobleached for 2000 ms (*t*_bl_) with a laser intensity of ~2 kW/cm^2^ (*I*_bl_). During 5,000 ms of recovery time, unbleached molecules re-enter the bleached area by Brownian motion; thereafter, a postbleach image is recorded in which individual molecules are distinguishable. The TOCCSL image was acquired with 5 ms excitation time (*t*_exp_) and laser intensity of 0.4–0.6 kW/cm^2^ (*I*_im_). The scale bar of the prebleach and postbleach image is 10 µm. ii) Timing protocol for a typical TOCCSL experiment, plotted as time *vs*. laser intensity. **b** Representative brightness distribution of the oligomeric fractions obtained from TOCCSL runs on ten cells, plotted as probability density function (PDF). **c** NET co-exists as monomers (~60%) and dimers (~40%) at the plasma membrane. Each data point represents an independent experiment with TOCCSL runs on 15 cells. Bars represent means ± SD.

Here, we examined the subunit stoichiometry of NET on a single-molecule level and assessed the effect of PIP_2_ on NET oligomerization and transporter kinetics. Single-molecule imaging is typically not feasible when fluorescently labeled proteins are expressed at high densities. To overcome this issue, we applied a method referred to as “thinning out clusters while conserving stoichiometry of labeling” (TOCCSL^42^, Fig. 1a) to determine the oligomeric state of NET. In TOCCSL, a rectangular area of the bottom cell membrane is irreversibly photobleached by a high-intensity laser pulse. Subsequently, unbleached molecules enter the bleached area by Brownian motion. During the onset of fluorescence recovery, individual fluorescence spots can be monitored. The brightness of these spots is then compared to the brightness of a single mGFP-NET molecule to determine the oligomeric distribution. To further elucidate the involvement of PIP_2_, we studied the putative PIP_2_ binding sites R121, K334, and R440, which correspond to R144, K352, and K460, respectively, in SERT^17^. Specifically, we mutated the residues to uncharged alanines with the aim of diminishing interactions with the negatively charged PIP_2_. Moreover, we studied the oligomerization of the NET variant A457P; this variant has been linked to orthostatic intolerance and oligomeric complexes between mutant and wildtype have been proposed as explanation for severe NET dysfunction in heterozygous subjects^8,43^. With this study, we therefore aimed to shed more light on processes that drive NET regulation in order to facilitate the development of novel therapeutic strategies for disease states and conditions related to NET. Examination of NET oligomerization, and the processes that regulate it, helps to comprehend the role of quaternary transporter arrangement in transporter function and stimulant drug action. We found evidence that PIP_2_ directly affects the quaternary organization of NET at the plasma membrane by stabilizing dimers. Our data furthermore suggest that PIP_2_ modulates substrate efflux but not uptake. Enhanced knowledge of NET regulation will therefore contribute to a better understanding of NET-associated diseases.

## Results

### NET molecules co-exist as monomers and dimers at the plasma membrane of live cells

**Fig. 2:**
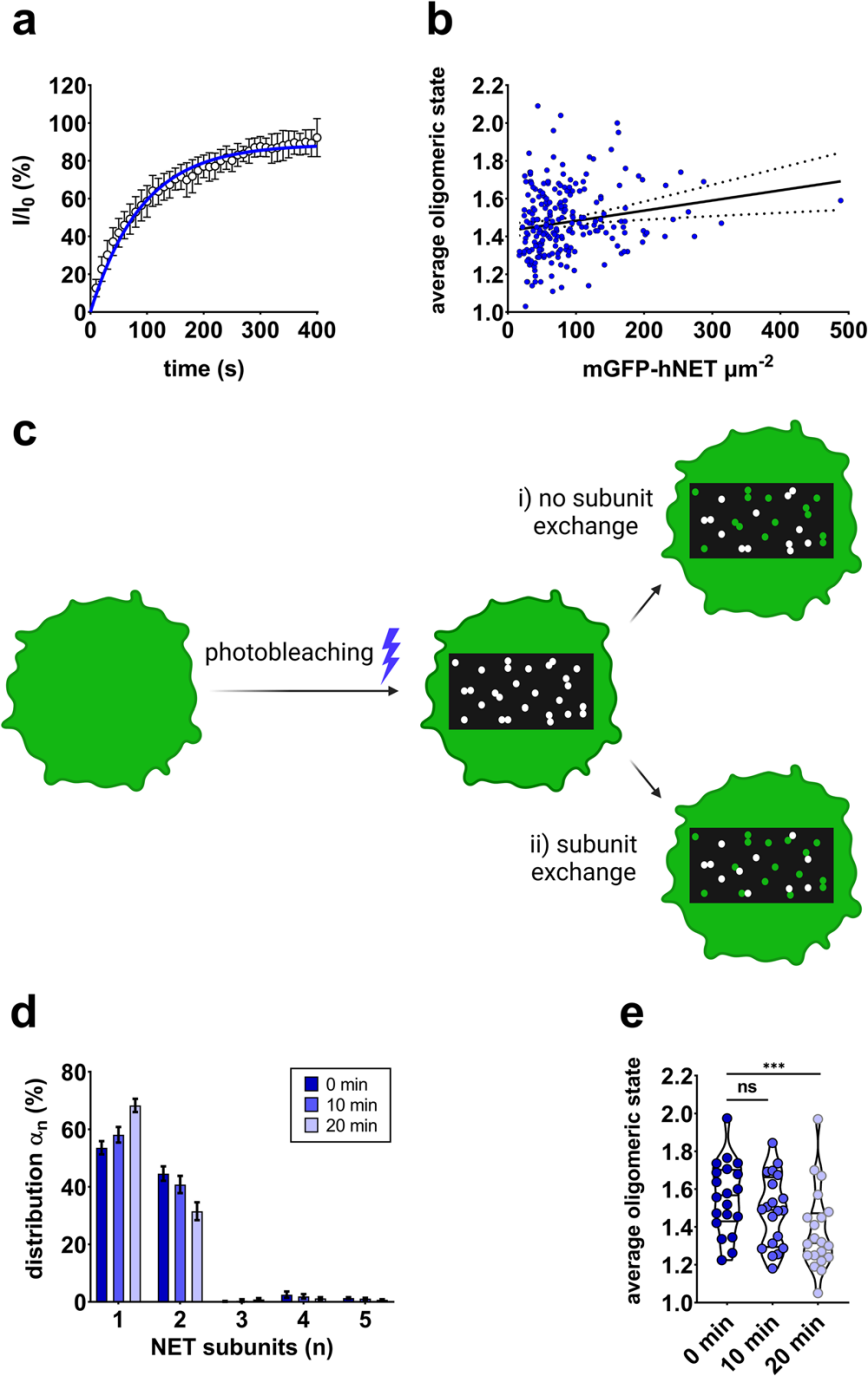
Mobile fraction, distribution, and stability of NET subunits. **a** Mobile fraction of NET determined by FRAP microscopy. Data points represent averages of ten cells (± SD), which were fitted by a one-phase association curve (Equ. 1). **b** NET oligomerization displays a slight transporter density-dependent behavior. Densities of 261 cells measured under control conditions were plotted against the average oligomeric state, resulting in a significant correlation (Spearman r: 0.1898, *P* < 0.01). **c** Sketch showing two potential scenarios. i) Scenario 1: dimers are stable; repeated runs result in a decreased number of active fluorophores but no altered distribution. ii) Scenario 2: dimers exchange subunits; the exchange of bleached (white) and unbleached (green) dimers results in an increasing fraction of apparent monomers. **d** Repeated TOCCSL runs on the same cell indicate that a small portion of NET dimers exchange subunits over time. A total of 20 cells were repeatedly measured three times over the course of 20 min. Data show means ± SD. **e** Average oligomeric state of NET molecules for each of the repeatedly measured cells.

The brightness distribution plotted as a probability density function (PDF) derived from TOCCSL experiments is shown in Fig. 1b, revealing the co-existence of monomers (~60%) and dimers (~40%) at the plasma membrane (Fig. 1c). Single-molecule experiments were performed on live Chinese hamster ovary (CHO) cells transfected with mGFP-hNET. Fluorescence recovery after photobleaching (FRAP) of ten cells yielded a mobile fraction of 89 ± 2% (95% CI) for mGFP-hNET molecules located at the plasma membrane (Fig. 2a). Analyzed by nonparametric Spearman correlation, the average oligomeric state correlated with transporter densities (Spearman r: 0.19, *P* [two-tailed] < 0.01; Fig. 2b). To assess whether wildtype NET dimers exchange subunits over time, 20 cells were repeatedly imaged 3 times in 10-min intervals, based on previous studies^17,36^. The following two scenarios could occur: i) if there were no subunit exchange, the same distribution would be obtained for each run, albeit with a decrease in the amount of active fluorophores as dimers get bleached during each run (Fig. 2c; i). ii) In case bleached and unbleached dimers would exchange subunits, the subunit distribution would shift to an increasing proportion of apparent monomers, which in part are dimers consisting of a bleached and an unbleached molecule (Fig. 2c; ii). The repeated TOCCSL experiments suggest that wildtype NET dimers do engage in subunit exchange (Fig. 2d). After 20 min, the apparent average oligomeric state was significantly decreased (Fig. 2e), indicating the presence of dimers consisting of a bleached and an unbleached molecule. The number of active fluorophores in the field of view was decreased by 64% and 80% after 10 and 20 min, respectively.

### PIP_2_ stabilizes NET dimers whereas cholesterol does not affect NET oligomerization

**Fig. 3:**
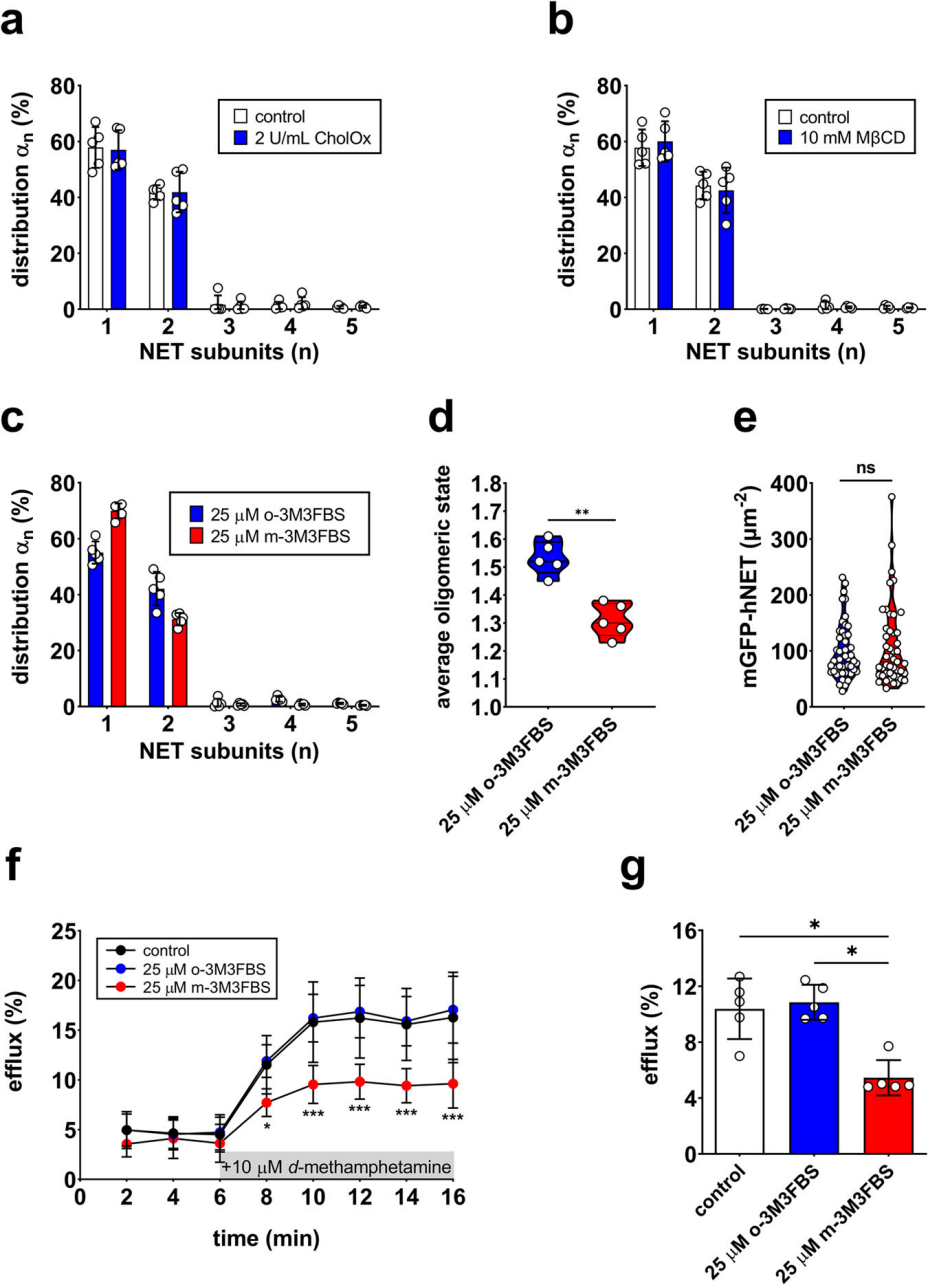
Effect of cholesterol and PIP_2_ on NET oligomerization and function. Oxidation (**a**) and depletion (**b**) of membrane cholesterol did not affect NET oligomerization. Each data point shows an independent experiment with TOCCSL runs on ten cells. Bars represent means ± SD. **c** NET oligomerization after PIP_2_ depletion with the direct PLC activator m-3M3FBS and after treatment with its inactive analog o-3M3FBS. Each data point represents an independent experiment with TOCCSL runs on ten cells. Bars represent means ± SD. **d** PIP_2_ depletion resulted in a significantly decreased average oligomeric state (**P < 0.01). **e** Surface density of mGFP-hNET after m-3M3FBS or o-3M3FBS pretreatment was statistically indifferent. **f** NET-mediated substrate efflux over time, induced with 10 µM *d*-methamphetamine after 6 min. The data were derived from five independent experiments (mean ± SD). Single data points are shown in Supplementary Fig. 6a. **g** Total *d*-methamphetamine-induced substrate efflux was decreased under PIP_2_-depleted conditions. Bars represent mean efflux (±SD) of five experiments, normalized to basal efflux.

To shed more light on the potential role of lipid plasma membrane components in wildtype NET oligomerization, plasma membrane cholesterol and PIP_2_ levels were manipulated. Cholesterol was either oxidized with 2 U/mL cholesterol oxidase (CholOx) or depleted with 10 mM methyl-β-cyclodextrin (MβCD). However, neither oxidation nor depletion of cholesterol affected wildtype NET oligomerization (Fig. 3a, b and Supplementary Fig. 1a–d). Additionally, the direct PLC activator m-3M3FBS was used to deplete the plasma membrane of PIP_2_. Compared to its inactive analogue o-3M3FBS, m-3M3FBS induced a noticeable but not complete dissociation of wildtype NET dimers (Fig. 3c), resulting in a statistically significant decrease in the average oligomeric state (Fig. 3d). Transporter densities at the plasma membrane were the same for both treatments, which excludes a difference in density as the explanation for the observed decrease in average oligomerization after m-3M3FBS treatment (Fig. 3e). The difference between both treatment conditions was more distinct at lower transporter densities (Supplementary Fig. 1e, f).

### Interactions of PIP_2_ with residues R121, K334, and R440 are not crucial for NET dimer stabilization

**Fig. 4:**
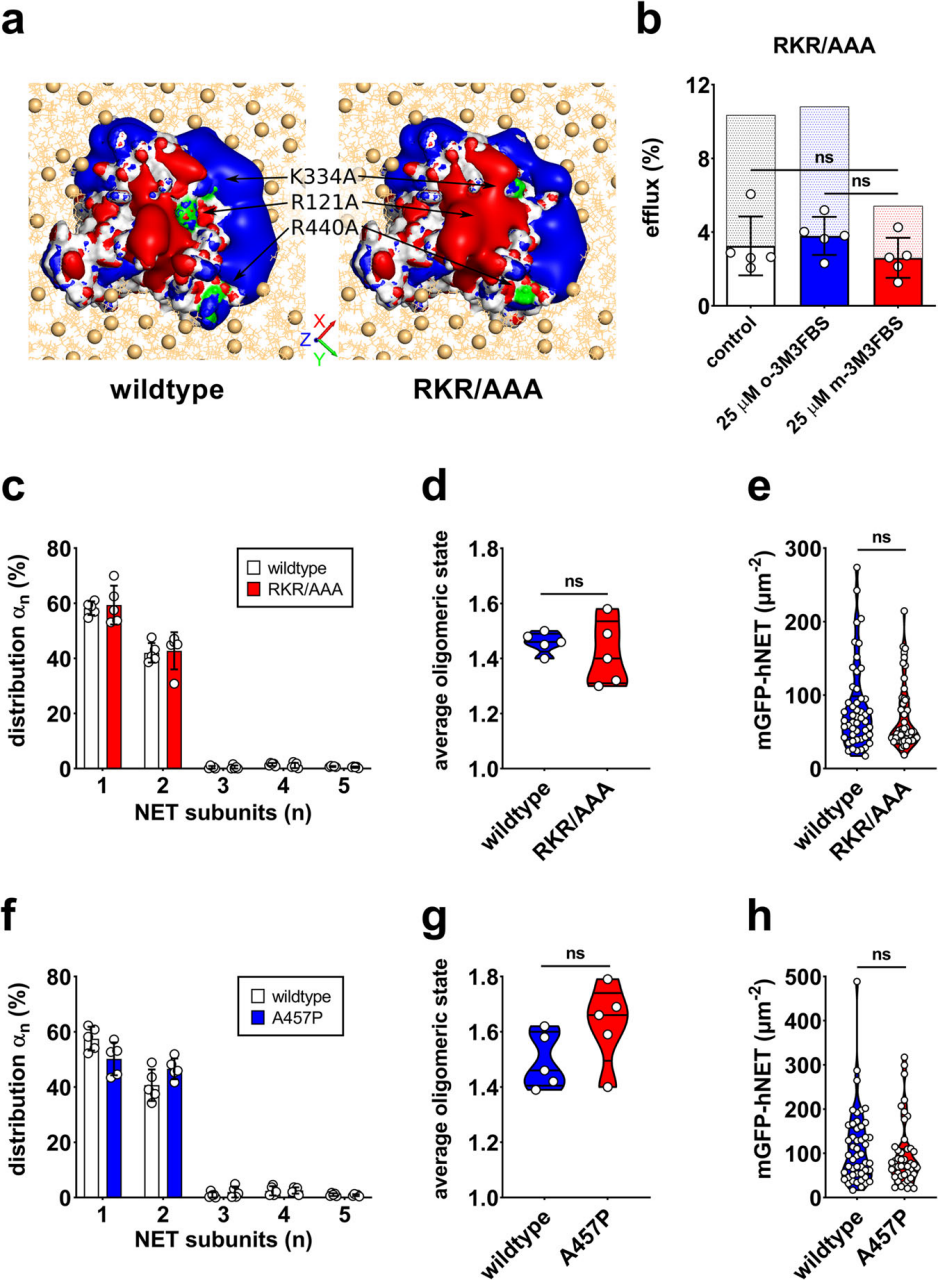
Subunit stoichiometry and function of the RKR/AAA and A457P mutant. **a** Electric field of the intracellular side of wildtype and mutant NET. The protein’s solvent-accessible surface is shown as white surface except for the residues replaced by the mutation, which are green. Red surface indicates the negative electric isopotential of -2 kT/e while blue surface shows the +2 kT/e. Possible protein-lipid interactions are depicted by showing the inner leaflet of a modeled POPC + cholesterol membrane indicated as orange lines and the phosphate headgroups as spheres. **b** Total *d*-methamphetamine-induced substrate efflux for the RKR/AAA mutant under PIP_2_-depleted conditions was statistically indifferent from control. Bars represent mean efflux (± SD) of five experiments, normalized to basal efflux. Efflux for wildtype NET is included as shaded bars for comparison. **c** Subunit distribution of the RKR/AAA mutant. Each data point represents an independent experiment with TOCCSL runs on ten cells. Bars represent means ± SD. **d** The average oligomeric state of the RKR/AAA mutant was indifferent from wildtype. **e** NET-RKR/AAA surface densities of the imaged cells did not differ from wildtype NET densities. **f** Subunit distribution of the A457P mutant. Each data point represents an independent experiment with TOCCSL runs on ten cells. Bars represent means ± SD. **g** The average oligomeric state of the A457P mutant was indifferent from wildtype. **h** NET-A457P surface densities of the imaged cells did not differ from wildtype NET densities.

To assess whether the NET residues R121, K334, and R440 stabilize NET dimers through binding to PIP_*2*_, they were mutated to uncharged alanine residues with the aim of diminishing interactions with the negatively charged PIP_2_. Intracellular retaining of NET-R121A/K334A/R440A (NET-RKR/AAA) was comparable to wildtype NET (Supplementary Figs. 2, 3) and importantly, also surface expression of cells used for imaging was the same (Fig. 4e). Compared to wildtype, the RKR/AAA mutant had a slightly decreased mobile fraction of 76% (95% CI: 74–78%). To get insight into how the RKR/AAA mutation may affect electrostatic interactions with PIP_2_, both NET model structures (wildtype and the RKR/AAA mutant) were analyzed using the adaptive Poisson-Boltzmann solver (APBS). The obtained electric field showed that the positive electric field around the protein’s intracellular side is only slightly affected by the mutation (Fig. 4a). In accordance with this finding, TOCCSL experiments revealed that the oligomerization of the RKR/AAA mutant is indifferent from wildtype (Fig. 4c–e).

### The orthostatic intolerance-associated NET variant A457P forms dimers

The NET variant A457P is associated with orthostatic intolerance and displays significantly decreased substrate transport and surface expression compared to wildtype^8,43^. The alanine to proline mutation is located on transmembrane helix 9 and causes a distinct change in the secondary structure^43^. Previous studies showed that NET-A457P co-immunoprecipitates with wildtype NET^43^. In the present study, we studied the oligomerization of the A457P mutant on a single-molecule level in live cells. The mobile fraction of the A457P mutant was 89% (95% CI: 88–91%) and therefore equal to wildtype. Despite a high proportion of NET-A457P being retained within the cell (Supplementary Fig. 4), the membrane expression was sufficient for TOCCSL runs in total internal reflection fluorescence (TIRF) mode. In accordance with previous co-immunoprecipitation studies, TOCCSL runs revealed that the A457P mutant forms dimers at the plasma membrane (Fig. 4f). Transporter densities of the imaged cells as well as the average oligomeric state of the A457P mutant were statistically indifferent from wildtype (Fig. 4g, h).

### PIP_2_ modulates NET-mediated substrate efflux but not uptake in vitro

PIP_2_ plays an important role in various cellular processes; among other effects, it modulates SERT-mediated substrate efflux^16^ and DAT-related amphetamine behavior (i.e., psychomotor and rewarding properties) in *Drosophila melanogaster*^18^. Currently, it is mostly unknown how PIP_2_ affects NET function. Pretreatment with 25 µM m-3M3FBS did not significantly alter single-point uptake, substrate saturation, or plasma membrane expression of wildtype NET when compared to pretreatment with o-3M3FBS (Supplementary Fig. 5a–d). Compared to wildtype, basal uptake of the RKR/AAA and A457P mutants was decreased by 64% and 95%, respectively (Supplementary Fig. 5a). As for wildtype, PIP_2_ depletion did not affect single-point uptake or substrate saturation of the mutants (Supplementary Fig. 5a, b). In contrast to substrate uptake, PIP_2_ depletion substantially decreased *d*-methamphetamine-induced substrate efflux of wildtype NET (Fig. 3f, g and Supplementary Fig. 6a). The RKR/AAA mutant displayed a 69% decreased efflux, which was not affected by PIP_2_ depletion (Fig. 4b and Supplementary Fig. 6b). These results are in line with studies showing that PIP_2_ depletion decreases substrate-induced efflux but not uptake in DAT^18^ and SERT^16^. The A457P mutant did not mediate any substrate efflux (Supplementary Fig. 6c). An overview of substrate efflux for all time points and conditions is provided in Supplementary Table 1. For the functional characterization of NET, we utilized human embryonic kidney (HEK)-hNET cells as done in previous studies^16,35,36^. PIP_2_ levels in mGFP-hNET-transfected CHO and HEK 293 cells were statistically indistinguishable, justifying the use of different cell lines (Supplementary Fig. 7).

## Discussion

NET regulates the cardiovascular sympathetic activity and is a key target of various psychoactive substances. Genetic or acquired deficits in NET-mediated norepinephrine inactivation may result in hyperadrenergic states. To advance our understanding of NET organization and function, this study examined NET oligomerization on a single-molecule level. Previous studies revealed differences between the oligomerization of DAT^36^ and SERT^17,35^. Namely, the two transporters differed regarding their subunit distribution. Here, we report the presence of NET dimers at the plasma membrane of live cells measured by single-molecule microscopy. A high degree of mobile NET molecules corroborates the applicability of the TOCCSL approach. Our results are in line with co-immunoprecipitation experiments showing the presence of NET homodimers^44^. We found that the subunit distributions of NET and DAT are virtually identical but differ from the distribution of SERT through the absence of higher-order oligomers. All monoamine transporters share a high degree of sequence identity but the two catecholamine transporters NET and DAT are the most similar with 72% sequence identity of their respective transmembrane domains^2,45^. An identical oligomeric pattern for DAT and NET is therefore not unexpected. Extensive molecular dynamics simulations revealed that all transmembrane helices of DAT can contribute to dimer interfaces except the bundle domain^46^. It is therefore possible that the same applies for NET dimer interfaces.

Cell membrane lipids including cholesterol and PIP_2_ play crucial roles in monoamine transporter oligomerization and function^15–20,36,41,47–49^. Still, the effects of cell membrane lipids on DAT and SERT oligomerization differ^17,19,36^. It is currently mostly unknown how cell membrane lipids affect NET organization and function. Plasma membrane cholesterol facilitates tighter packing of the hydrocarbon chains, resulting in lower lateral mobility of lipids and an increase in membrane thickness^49,50^. Perturbation of membrane cholesterol may result in distinct changes in the organization of clustered proteins or the dimerization of mobile components^51^. Moreover, membrane cholesterol modulates conformation, pharmacology, and kinetics of monoamine transporters^47,48,52^. Hence, we manipulated cholesterol levels to examine whether cholesterol-rich liquid-ordered membrane phases are involved in the formation of NET dimers. However, our study showed that neither oxidation nor depletion of membrane cholesterol affects NET dimerization. Whereas cholesterol levels do not contribute to oligomer stabilization for either NET or DAT, the most striking difference between NET and DAT oligomerization is the stabilization of NET dimers by PIP_2_. Nonetheless, in contrast to SERT oligomerization^17^, the residual exchange of NET subunits suggests that PIP_2_ does not completely kinetically trap NET dimers at the plasma membrane. Rather, NET oligomerization at the cell membrane resembles the behavior of SERT oligomers at the endoplasmic reticulum^17^. It needs to be considered that the evanescent field of TIRF illumination penetrates into the sample to a depth of about 100 nm; thereby, it potentially excites fluorophores at the endoplasmic reticulum in addition to those at the cell membrane (Supplementary Fig. 8). Compared to SERT, the proportion of intracellularly retained NET, and therefore the contribution of endoplasmic NET to the observed results, may be distinctively higher. Furthermore, it needs to be considered that the degree of subunit exchange might be slightly overestimated due to partial bleaching of dimers during repetitive runs. Still, previous studies indicate that partial bleaching does not substantially affect the results^17,36^.

Altered norepinephrine and PIP_2_ signaling have both been associated with neurological and neuropsychiatric disorders^53,54^. Here, we provide evidence that PIP_2_ modulates NET organization and function. For a better understanding of the importance of PIP_2_ for NET function, we assessed its role in NET-mediated substrate uptake and efflux. Previous studies showed that PIP_2_ affects DAT and SERT-mediated substrate efflux but not substrate uptake^16,18,20^. Similarly, our in vitro results show that PIP_2_ does not affect substrate uptake by NET, while PIP_2_ depletion affects NET-mediated substrate efflux. As m-3M3FBS decreases only a part of the membrane PIP_2_^16^, it remains unresolved whether complete depletion of PIP_2_ would further decrease NET-mediated efflux. As alternative approach to pharmacological PIP_2_ depletion, we aimed to reduce interactions of PIP_2_ with potential binding sites of NET by mutation. For SERT, we have previously identified residues K352 and K460, and to a lesser extent R144, as PIP_2_ binding sites involved in the stabilization of SERT higher-order oligomers^17^ and in substrate-induced efflux^16^. Similarly, the electrostatic interaction of PIP_2_ with R443 in DAT, which corresponds to K460 in SERT, has been shown to regulate dopamine efflux^18^. We therefore focused on the NET residues R121, K334, and R440, which correspond to R144, K352, and K460, respectively, in SERT. We mutated the positively charged residues of interest to uncharged alanines, thereby diminishing interactions with the negatively charged PIP_2_. The RKR/AAA mutant displayed a decreased efflux; yet, the oligomerization of wildtype NET and the RKR/AAA mutant was indifferent. We used electrostatic field analysis to better understand the interaction of PIP_2_ with R121, K334, and R440 in NET. A triple mutation of analogous residues to alanines resulted in a distinct change of the electrostatic potential in SERT, reducing the attractive force for PIP_2_ binding^16^. For NET, our electrostatic field analysis suggests that the electrostatic field remains relatively unchanged and the remaining positively charged residues located at the inner side of the transporter can compensate the effect of the mutation to a certain degree (Fig. 4a). Specifically, the residues K61, K62, K254, K257, K261, R341, K443, R512, R518, and K522 all contribute to the positively charged rim that may interact with PIP_2_. This suggests that the RKR/AAA mutant and wildtype NET are similarily sensitive to PIP_2_ depletion. Despite the small change in the electrostatic field, substrate efflux mediated by the RKR/AAA mutant was substantially lower than for wildtype NET. Moreover, in contrast to wildtype, PIP_2_ depletion did not significantly affect substrate efflux for the RKR/AAA mutant. Taken together, this indicates that reduced interactions of the RKR/AAA mutant with PIP_2_ are, at least in part, the cause of the decreased efflux. This therefore strengthens the hypothesis that the residues R121, K334, and R440 engage in PIP_2_ binding, thereby supporting specific NET conformations required for substrate-induced efflux.

Oligomeric complexes between wildtype NET and the disease variant A457P have been hypothesized to account for severe NET dysfunction in heterozygous subjects. The alanine to proline missense mutation in transmembrane helix 9 adds a kink into the secondary structure of NET and has been linked to orthostatic intolerance^8,43^. NET-A457P displays an overall reduced surface expression and a drastically reduced substrate transport^43^. The oligomerization of the A457P variant has so far not been studied. In accordance with previous studies^43^, we measured substantially reduced substrate uptake and consequently no *d*-methamphetamine-induced efflux. Importantly, our study revealed that at comparable transporter densities, the subunit distribution of the A457P mutant does not deviate from wildtype. Hence, this fuels the hypothesis that oligomeric complexes between wildtype NET and the A457P mutant are the underlining mechanism for severe NET dysfunction in heterozygous subjects suffering from orthostatic intolerance.

The physiological role of NET oligomerization still remains enigmatic. Transporter function is affected by many different factors and the contribution by the quaternary structure of the transporter remains unclear. NET oligomerization may pose a mechanism to modulate norepinephrine efflux induced by sympathomimetic agents or to control NET trafficking. Future research should focus on the different biophysical properties of NET dimers compared to monomers (e.g., bulkiness and slower diffusion) and their role in the spatial arrangement of NET.

In summary, we show that PIP_2_ affects the cardiovascular system by modulating the quaternary organization and efflux function of NET in addition to regulating vascular ion channels. Altered norepinephrine and PIP_2_ signaling have been implicated in various neurological and neuropsychiatric diseases. Linking PIP_2_ to NET organization and function provides further insights into disease etiology. NET oligomerization shares some similarities and some differences with the other monoamine transporters. Differences exist in the subunit stoichiometry and the stability of the individual transporter oligomers. The two catecholamine transporters NET and DAT both form monomers (~60%) and dimers (~40%) at the plasma membrane; however, compared to DAT, NET dimers are more dynamic and exchange subunits on the time scale of minutes. This manifests in subunit exchange, transporter density dependence, and dimer dissociation under PIP_2_-depleted conditions. SERT is the only monoamine transporter that forms higher-order oligomers, which are stabilized by PIP_2_ as well. The present study therefore demonstrates that the oligomerization behavior of each monoamine transporter has unique features. Unlike its effect on oligomerization, the effect of PIP_2_ on transporter kinetics is similar for all monoamine transporters. Transporter-mediated substrate efflux is modulated through interactions of PIP_2_ with positively charged residues on intracellular loop 4; decreased PIP_2_ levels result in decreased efflux.

## Material and Methods

### Materials availability

Plasmids generated in this study have been made available at addgene.org and carry the following IDs: 193364 (mGFP-hNET), 193365 (mGFP-hNET- A457P), and 193366 (mGFP-hNET- R121A/K334A/R440A).

### Plasmids

A monomeric version of green fluorescent protein (mGFP; FPbase ID: QKFJN) was prepared by A206K mutation of enhanced GFP^35,55^. Human NET was cloned to a vector expressing mGFP via EcoRI/BamHI, resulting in a fusion protein of hNET with mGFP linked to the N-terminus. The A457P and R121A/K334A/R440A mutations were introduced into the mGFP-hNET plasmid using the Quikchange Lightning Site-Directed Mutagenesis Kit (cat#: 210518, Agilent Technologies, Santa Clara, CA, USA). The following primers were obtained from Microsynth AG (Balgach, Switzerland): forward primer A457P: 5’-CAGCACTTTCCTTCTCCCCCTGTTCTGCATAAC-3’; reverse primer A457P: 5’-GTTATGCAGAACAGGGGGAGAAGGAAAGTGCTG-3’; forward primer R121A: 5’-TGGGACAGTACAACGCGGAGGGGGCTGCC-3’; reverse primer R121A: 5’-GGCAGCCCCCTCCGCGTTGTACTGTCCCA-3’; forward primer R334A: 5’-TGATTGCATTTGCCAGTTACAACGCATTTGACAACAACTGTTACAGGG-3’; reverse primer R334A: 5’-CCCTGTAACAGTTGTTGTCAAATGCGTTGTAACTGGCAAATGCAATCA-3’; forward primer R440A: 5’-CTTCCAGGTCCTGAAGGCACACCGGAAACTCTTC-3’; reverse primer R440A: 5’-GAAGAGTTTCCGGTGTGCCTTCAGGACCTGGAAG-3’. All plasmids were sequenced before use.

### Cell culture

CHO cells (cat#: 85050302, Sigma-Aldrich, Vienna, Austria) were cultured at 37 °C and 5% CO_2_ in Dulbecco′s Modified Eagle′s Medium/Nutrient Mixture F-12 Ham (cat#: D8437, Sigma-Aldrich) supplemented with 10% fetal bovine serum (FBS; cat#: F7524, Sigma-Aldrich), 100 U/mL penicillin, and 100 µg/mL streptomycin (cat#: P4333, Sigma-Aldrich). HEK 293T cells (cat#: HCL4517, Thermo Fisher Scientific, Vienna, Austria) were cultured at 37 °C and 5% CO_2_ in Dulbecco′s Modified Eagle′s Medium (4.5 g/L glucose and 584 mg/L L-glutamine [cat#: L0102, Biowest, Nuaillé, France]) supplemented with 10% FBS (cat#: FBS-11A, Capricorn Scientific, Ebsdorfergrund, Germany), 100 U/mL of penicillin, and 100 µg/mL of streptomycin. Cell lines were regularly tested for mycoplasma contamination using MycoAlert Mycoplasma Detection Kit and MycoAlert Assay Control Set (cat#: LT07-418 and LT07-518, respectively, Lonza, Basel, Switzerland).

### Transfections

The plasmids were transfected into CHO cells using TurboFect Transfection Reagent (cat#: R0531, Thermo Fisher Scientific). 24 h prior to transfection, 80,000 cells in 4 mL culture medium were seeded into a well of a 6-well plate. 4 µg of DNA were diluted in 400 µL of reduced serum medium (Gibco OptiMEM; cat#: 31985062, Thermo Fisher Scientific) and 8 µL of transfection reagent were added. After 20 min incubation at room temperature, the mixture was added dropwise to the cells. The plate was gently rocked and then incubated at 37 °C and 5% CO_2_ for 48 h. Thereafter, the culture medium was replaced with medium containing the selection antibiotic G418 (800 µg/mL; cat#: G418-B, Capricorn Scientific). After ten days, the cells were frozen until further use. Uptake experiments were performed in HEK 293T cells transiently transfected with polyethyleneimine (linear, molecular weight: ~25,000; cat#: sc-507213, Santa Cruz Biotechnology, Dallas, TX, USA). 2,500,000 cells were seeded into a 10-cm cell culture dish. The following day, 8 µg DNA and 24 µg polyethyleneimine were diluted in 1 mL serum-free growth medium, vortexed, and incubated for 15 min at room temperature. The mixture was then added to the cells.

### Sample preparation

For single-molecule and FRAP microscopy experiments, 8-chamber Nunc Lab-Tek #1.0 borosilicate coverglass systems were used (cat#: 155411, Thermo Fisher Scientific). The chambers were sterilized with 70% isopropanol and coated with fibronectin bovine plasma (cat#: F1141, Sigma-Aldrich) for 20 min at 37 °C. Thereafter, the chambers were washed twice with phosphate-buffered saline (cat#: D8537; Sigma-Aldrich). The cells were detached with accutase solution (cat#: A6964, Sigma-Aldrich) for 4 min at 37 °C; 50,000 cells resuspended in 500 µL cell culture medium were then seeded into each well. After 12–16 h, the cell culture medium was replaced with imaging buffer (2% FBS in Hanks’ Balanced Salt Solution [cat#: H8264, Sigma-Aldrich]) and the samples were measured.

For confocal microscopy, transfected CHO cells were seeded into a poly-D-lysine-coated (0.05 mg/mL; cat#: P1149, Sigma-Aldrich) 29-mm glass-bottom dish with 20-mm micro-well #1.5 cover glass (cat#: D29-20-1.5-N, Cellvis, Mountain View, CA, USA) at a density of 100,000 cells per dish. The following day, the culture medium was removed and the cells were incubated for 10 min with 10 nM of the nisoxetine-based fluorescent probe AC1-146^56^ diluted in Krebs-HEPES buffer (KHB: 25 mM HEPES, 120 mM NaCl, 5 mM KCl, 1.2 mM CaCl_2_, 1.2 mM MgSO_4_, and 5 mM D-glucose, pH 7.3) at room temperature.

### Cholesterol oxidation and depletion

For membrane cholesterol oxidation, the samples were incubated with 2 U/mL cholesterol oxidase (cat#: C8649, Sigma-Aldrich) dissolved in imaging buffer for 30 min at 37 °C. For cholesterol depletion, the samples were incubated with 10 mM MβCD (cat#: C4555, Sigma-Aldrich) in Hank’s Balanced Salt Solution for 30 min at 37 °C. After incubation, the samples were transferred to the microscope and measured immediately without the replacement of the incubation buffer.

### PIP_2_ depletion

PIP_2_ levels were depleted with the specific PLC activator 2,4,6-trimethyl-*N*-[3-(trifluoromethyl)phenyl]benzenesulfonamide (m-3M3FBS; cat#: T5699, Sigma-Aldrich). For single-molecule microscopy, the cells were either incubated with 25 µM of m-3M3FBS or its inactive ortho analog o-3M3FBS (cat#: sc-204142, Santa Cruz Biotechnology) for 20 min at 37 °C in imaging buffer. After incubation, the samples were transferred to the microscope and measured immediately without the replacement of the incubation buffer. For radiotracer experiments, the cells were incubated with 25 µM m-3M3FBS or o-3M3FBS for 10 min.

### Single-molecule microscopy

Single-molecule microscopy was conducted based on earlier studies^17,35,36^. Fluorophores were excited with 488-nm light originating from a directly modulated diode laser (LBX-488, installed in L6Cc laser combiner; Oxxius, Lannion, France). The illumination intensity and timing were adjusted using custom-written software in LabVIEW (National Instruments, Austin, TX, USA). The laser beam was focused onto the back-focal plane of a Plan-Apochromat objective (100x/1.46 NA; Zeiss, Jena, Germany) mounted on an inverted Zeiss Axiovert 200 microscope. The emission light was filtered using appropriate emission filter sets (FF01-538/685-25; Semrock, Rochester, NY, USA, and zt488/640rpc; Chroma, Bellows Falls, VT, USA) and imaged onto a back-illuminated charge-coupled device camera (LNCCD1300-PB, Roper Scientific, Planegg, Germany), cooled to -110 °C with liquid nitrogen. A slit aperture (Owis, Staufen im Breisgau, Germany) was used to restrict the excitation and photobleaching area. Stroboscopic illumination with excitation times of 5 ms (*t*_exp_) was used. The samples were excited and bleached in TIRF mode. All experiments were performed at room temperature.

### Fluorescence recovery after photobleaching

The mobile fraction of mGFP-hNET and both mutants was assessed by FRAP. An area of ~50 µm^2^ of the bottom plasma membrane was photobleached in TIRF mode and fluorescence recovery was measured every 10 s during a total recording time of 400 s. Data were fitted by a one-phase association curve (Equ. 1), where *I*_0_ represents the fluorescence signal before photobleaching, *I(t)* the fluorescence signal at time *t*, *mf* the mobile fraction, and *K* the recovery rate constant.

1 $$I(t)/{I}_{0}={mf}\times (1-{e}^{-{Kt}})$$

### Thinning out clusters while conserving stoichiometry of labelling (TOCCSL)

The TOCCSL protocol was adapted from previous studies^17,35,36^. For each run, a prebleach image was recorded and used to assess the surface density of mGFP-hNET. 50 ms after recording the prebleach image, an aperture-confined region of the bottom plasma membrane was photobleached for 2,000 ms (*t*_bl_) with a laser intensity of ~2 kW/cm^2^ (*I*_bl_). After a recovery time of 5,000 ms, the TOCCSL image was acquired with an excitation laser intensity of 0.4–0.6 kW/cm^2^ (*I*_im_). To assess the brightness of a single mGFP-hNET molecule, cells were repeatedly bleached for 200 ms with a laser intensity of ~2 kW/cm^2^; this substantially reduced the amount of active fluorophores, so that each NET oligomer contained a maximum of one active fluorophore^35^. These individual signals can be assumed to represent a pure monomeric fraction and were used for calculating the single mGFP brightness distribution. All laser intensities were determined in epifluorescence configuration. To assess whether NET oligomerization depends on transporter density, the transporter density of a total of 261 cells was plotted against the average oligomeric state of NET. The transporter density was calculated using the average fluorescence brightness of the prebleach image divided by the single-molecule brightness. To assess whether different NET dimers exchange subunits, three repetitive TOCCSL runs were performed on the same cell in 10-min intervals. The average oligomeric state of NET molecules of each cell after 10 and 20 min was compared to start conditions.

### Single-molecule brightness analysis

TOCCSL images were analyzed in MATLAB (Mathworks, Portola Valley, CA, USA) using an in-house algorithm. The pixel counts were converted to photon counts by offset subtraction and multiplying with the inverse gain (as per specification of the Roper camera). The individual diffraction-limited fluorescent signals from the regions of interest were fitted by a Gaussian function. The fitting routine yielded the single spot brightness B, which was then used to determine the oligomeric state of the mGFP–labeled constructs. The brightness values of all single spot signals obtained in the TOCCSL images were then plotted as a probability density function, *ρ*(B). Using autoconvolution, the monomer brightness distribution *ρ*_1_(B) was used to calculate the brightness distributions for dimers, *ρ*_2_(B), and for higher-order oligomers. The overall single spot brightness distribution *ρ*(B) was then fitted by a linear combination of *ρ*_1_(B), *ρ*_2_(B), and higher-order oligomers (Equ. 2).

2 $$	\rho \left({{{{{\rm{B}}}}}}\right)=\mathop{\sum }\limits_{n=1}^{{n}_{{\max }}}{\alpha }_{n}\times {\rho }_{n}(B) \\ 	{\mbox{with normalization}} \mathop{\sum }\nolimits_{n=1}^{{n}_{{\max }}}{\alpha }_{n}=1$$

Fitting *ρ*(B) yielded the fractions α_*n*_ of the different oligomeric states of co-diffusing transporter molecules with active mGFP molecules. A bootstrapping method was applied to calculate the standard deviations of each analysis. For this, random subsamples containing 50% of the data were analyzed in 100 repetitions.

### Confocal microscopy

Confocal images were recorded to assess the proportion of cell membrane expression of wildtype NET and mutants. The cell sample dish was mounted above a 60x oil immersion objective on a Nikon A1R+ laser scanning confocal microscope system (Nikon, Minato City, Tokyo, Japan). Images were acquired using a resonant scanner. The nisoxetine-based fluorescent probe AC1-146 and trypan blue solution were excited by a 561-nm laser line; mGFP was excited by a 488-nm laser line. A 525/50 nm and a 595/50 nm emission filter were used. The emitted light was collected with a high-sensitivity GaAsP detector. To assess NET internalization after PIP_2_ depletion, the ratio between plasma membrane fluorescence (F_PM_) and cytosolic fluorescence (F_Cyt_) was determined with ImageJ software^57^. The regions of interest (ROIs) were drawn in the 561-nm channel where the stained plasma membrane was clearly visible. Total fluorescence was calculated by multiplying the area and average fluorescence of each ROI in the 488-nm channel after background subtraction.

### Radiotracer experiments

Substrate saturation was determined in HEK 293T cells transiently transfected with mGFP-hNET. The cells were washed once with phosphate-buffered saline and detached with Trypsin-EDTA solution (cat#: T3924, Sigma-Aldrich). 24 h prior to the experiment, the cells were seeded onto poly-D-lysine-coated 96-well culture plates at a density of 200,000 cells/mL and grown as monolayers. After pretreatment with 25 µM m-3M3FBS, 25 µM o-3M3FBS, or vehicle control for 10 min, the cells were incubated in 50 μL of KHB containing 0.3–100 μM 1-methyl-4-phenylpyridinium (MPP^+^; cat#: D048, Sigma-Aldrich) for 3 min at room temperature. The dilution row was prepared by mixing various concentrations of non-tritiated MPP^+^ with 20 nM [^3^H]MPP^+^ (80–85 μCi mmol^−1^; American Radiolabeled Chemicals, St. Louis, MO, USA). Unspecific uptake was determined in the presence of 50 µM GBR12909 (cat#: D052, Sigma-Aldrich). Afterwards, 200 μL of Ultima Gold XR scintillation cocktail (cat#: 6013329, PerkinElmer, Waltham, MA, USA) was added, the plates were shaken, and tritiated substrate uptake was determined with a Wallac 1450 MicroBeta TriLux liquid scintillation counter (GMI, Ramsey, MN, USA). In addition to substrate saturation curves, single-point uptake was assessed as follows: cells expressing the protein of interest were washed with 200 μL of KHB and treated with 25 μM m-3M3FBS, 25 μM o-3M3FBS, or vehicle control for 10 min. Uptake was then assessed by exposing the cells to 20 nM [^3^H]MPP^+^ for 3 min. Non-specific uptake was determined in the presence of 50 µM GBR12909.

To assess NET-mediated substrate efflux, transporter-transfected cells were seeded onto poly-D-lysine coated 96-well plates at a density of 60,000 cells per well in a final volume of 200 µL. After 24 h, the cells were preloaded with 50 nM [^3^H]MPP^+^ dissolved in KHB for 20 min at 37 °C. Subsequently, the cells were washed three times with KHB and equilibrated for 10 min. Thereafter, the cells were incubated with 25 µM m-3M3FBS, 25 µM o-3M3FBS, or vehicle control for 10 min. Basal efflux was assessed during 6 min before substrate-induced efflux was initiated with 10 µM *d*-methamphetamine (cat#: M8750, Sigma-Aldrich) for 10 min. The supernatant was transferred to another 96-well plate and 200 µL Ultima Gold XR scintillation cocktail were added to the cells and supernatant. Radioactivity in the cells and supernatant was assessed with a Wallac 1450 MicroBeta TriLux Liquid Scintillation Counter. [^3^H]MPP^+^ release was expressed as percentage of the total radioactivity in cells and supernatant normalized to the basal efflux. Nonspecific release was determined in the presence of 30 µM nisoxetine (cat#: N151, Sigma-Aldrich).

### In silico modelling

As the structure of hNET is currently not known, we created a model based on the homologous outward-open hSERT crystal structure (PDB ID: 5I71)^58^, which has a sequence identity of 54% for the transmembrane domain. Homology modelling was performed with MODELLER 9.20^59,60^. Two sodium ions as well as a chloride ion were placed by MODELLER to their respective binding sites. Based on the available structural information and previous experience with SERT and DAT models, the glutamate residue 488 was protonated. The best model based on the DOPE score was used as wildtype and mutated (R121A, K334A, and R440A) to obtain the RKR/AAA mutant model. The homology models were used as the input for electrostatic field analysis using the adaptive Poisson-Boltzmann solver (APBS)^61,62^. Cytoplasmic and side view of a homology model of the RKR/AAA mutant are provided in Supplementary Fig. 9.

### Mass spectrometry

Phosphatidylinositol phosphates were prepared as reported by Clark and co-workers^63^. In short, lipids were extracted by an acidic Folch extraction and derivatized employing TMS-diazomethane. After removal of excess reagent, methylated 18:0-20:4 PI(4,5)P_2_ was measured employing an untargeted lipidomics workflow in positive polarization mode on a Bruker timsTOF Pro equipped with a VIP-HESI source (Bruker Corporation, Billerica, MA, United States). The frontend was a Thermo Fisher Scientific Vanquish H UHPLC with a Waters Acquity BEH C18 column (150 mm × 1 mm × 1.7 µm; Waters Corporation, Milford, MA, USA). Mobile phase A was 60% acetonitrile in 20 mM ammonium formate; mobile phase B was 10% acetonitrile in isopropanol supplemented with 10 mM ammonium formate. The following gradient was employed at a constant flow rate of 70 µL/min and 40 °C: 0 min; 2% B, 7 min; 2% B, 20 min; 100% B, 25 min; 100% B, followed by 5 min re-equilibration to starting conditions. 18:0-20:4 PI(4,5)P_2_ was quantified on MS1 level employing Bruker Compass DataAnalysis Version 5.3. Mass-to-charge, retention time, and CCS were matched to an authentic standard (18:0-20:4 PI[4,5]P_2_; cat#: 850165, Avanti Lipids, Birmingham, AL, USA).

### Statistics and reproducibility

Data were fitted and statistically analyzed with GraphPad Prism (version 9.3.1, San Diego, CA, USA). Statistical significance was determined as *P* < 0.05. To determine the subunit stoichiometry of NET, 10–15 cells per condition and day were measured on 5 different days. Average oligomerization under the different conditions was compared using a two-tailed Mann-Whitney test. A total of 261 cells were measured to assess whether NET dimerization depends on transporter density. Two-tailed nonparametric Spearman correlation was computed to assess the correlation between the average oligomeric state and transporter density. 20 cells were repeatedly measured to examine subunit exchange of NET dimers. The average oligomeric state of NET molecules of each cell after 10 and 20 min was compared to start conditions by the Friedman test followed by Dunn’s multiple comparison posthoc test. Batch release experiments were compared using the Mann-Whitney test followed by Dunn’s multiple comparison posthoc test. Substrate-induced efflux was assessed in five independent experiments performed in duplicate. Efflux curves were compared by two-way ANOVA, corrected for multiple comparisons using the Holm-Sidak method. Uptake was assessed in three independent experiments performed in triplicate. Single-point uptake after treatment with m-3M3FBS was compared to o-3M3FBS and vehicle control using the Kruskal-Wallis test followed by Dunn’s multiple comparison posthoc test. To assess NET internalization after PIP_2_ depletion, the ratio between mGFP fluorescence at the plasma membrane (F_PM_) and cytosolic fluorescence (F_Cyt_) of 30 cells was compared with a two-tailed Student’s t-test. PIP_2_ levels in CHO and HEK 293T cells were assessed in quadruplicate and normalized to cellular PI levels. Peak ratios were compared using a two-tailed Mann-Whitney test.

### Reporting summary

Further information on research design is available in the Nature Portfolio Reporting Summary linked to this article.

## Supplementary information

Supplementary Information

Reporting Summary

## Data Availability

Data supporting the findings of this study are available within the article and supplementary information and at zenodo.org upon reasonable request (Zenodo record: 7303693).

## Code availability

The MATLAB source code for TOCCSL image analysis can be accessed at: https://github.com/schuetzgroup/TOCCSL_analysis.
