# Supplementary material for: Phosphatidylinositol 4,5-bisphosphate (PIP2) facilitates norepinephrine transporter dimerization and modulates substrate efflux
Source: Commun Biol. 2022 Nov 17;5:1259. doi: 10.1038/s42003-022-04210-1 (PMC9672106; doi:10.1038/s42003-022-04210-1)
Supplement: Supplementary file 1 — Supplementary Information [file 42003_2022_4210_MOESM1_ESM.pdf]

Supplementary Information for

Phosphatidylinositol 4,5-bisphosphate (PIP_2) facilitates norepinephrine transporter dimerization and modulates substrate efflux

Dino Luethi[1,2], Julian Maier[1], Deborah Rudin[1], Dániel Szöllősi[1], Thomas J. F. Angenoorth[1], Stevan Stankovic[1], Matthias Schittmayer[3], Isabella Burger[3], Jae-Won Yang[1], Kathrin Jaentsch[1], Marion Holy[1], Anand Kant Das[2,4], Mario Brameshuber[2], Gisela Andrea Camacho-Hernandez[5], Andrea Casiraghi[5,6], Amy Hauck Newman[5], Oliver Kudlacek[1], Ruth Birner-Gruenberger[3,7], Thomas Stockner[1], Gerhard J. Schütz[2,#,*], and Harald H. Sitte[1,#,*]

Affiliations

[1] Institute of Pharmacology, Center for Physiology and Pharmacology, Medical University of Vienna, Waehringer Strasse 13A, 1090 Vienna, Austria

[2] Institute of Applied Physics, TU Wien, Lehargasse 6, 1060 Vienna, Austria

[3] Institute of Chemical Technologies and Analytics, TU Wien, Getreidemarkt 9, 1060 Vienna, Austria

[4] Physics Program, New York University Abu Dhabi, Saadiyat Island, 129188, Abu Dhabi, United Arab Emirates

[5] Medicinal Chemistry Section, Molecular Targets and Medications Discovery Branch, National Institute on Drug Abuse – Intramural Research Program, Baltimore, MD 21224, USA.

[6] Department of Pharmaceutical Sciences, University of Milan, Via Luigi Mangiagalli 25, 20133, Milan, Italy

[7] Diagnostic and Research Institute of Pathology, Medical University of Graz, Neue Stiftingtalstrasse 6, 8010 Graz, Austria

[*] To whom correspondence may be addressed. Email: schuetz@iap.tuwien.ac.at

[*] To whom correspondence may be addressed. Email: harald.sitte@meduniwien.ac.at

This PDF file includes:

Supplementary Figs. 1 to 9

Supplementary Table 1

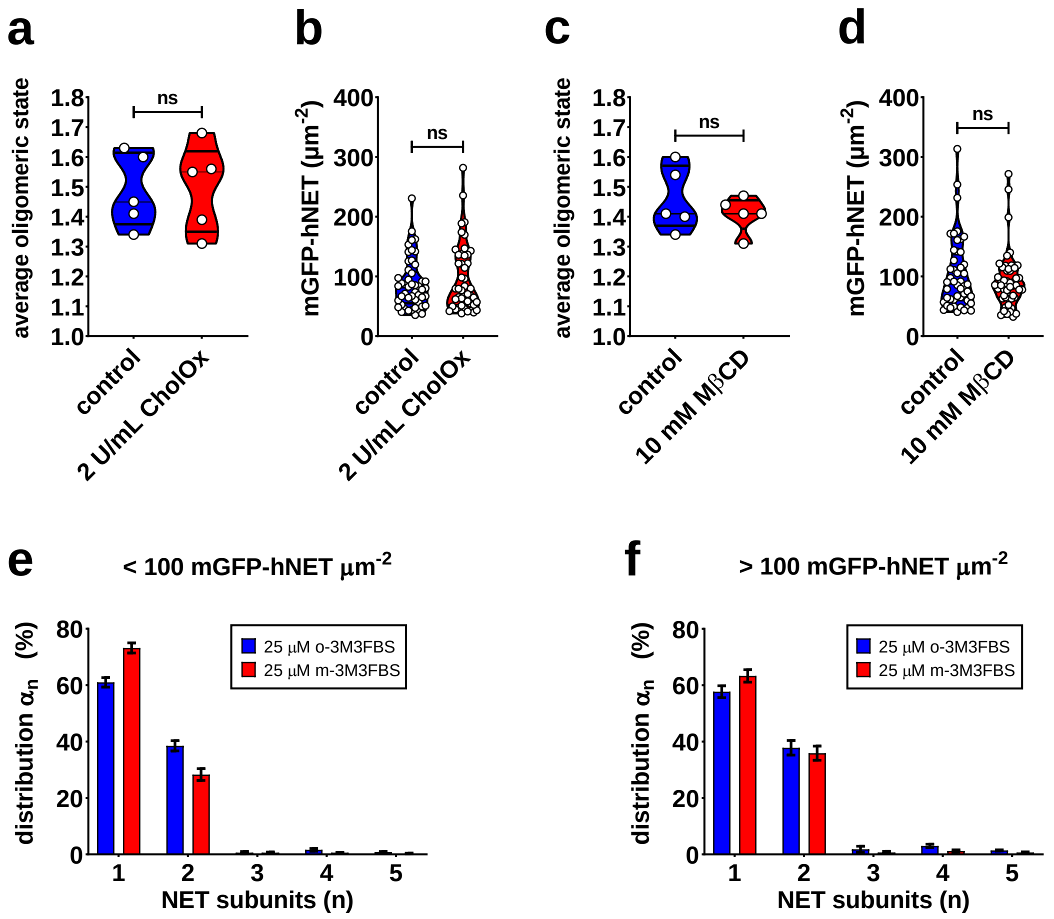

Supplementary Fig. 1. PIP_2 depletion affects NET oligomerization stronger at lower transporter densities, while cholesterol oxidation and depletion have no effect. Average oligomeric state and transporter densities were statistically indifferent after cholesterol oxidation (a and b) and after cholesterol depletion (c and d). NET oligomerization was more affected by PIP_2 depletion at densities of < 100 (e) compared to > 100 (f) transporter molecules per μm^2. Bars represent means ± SD.

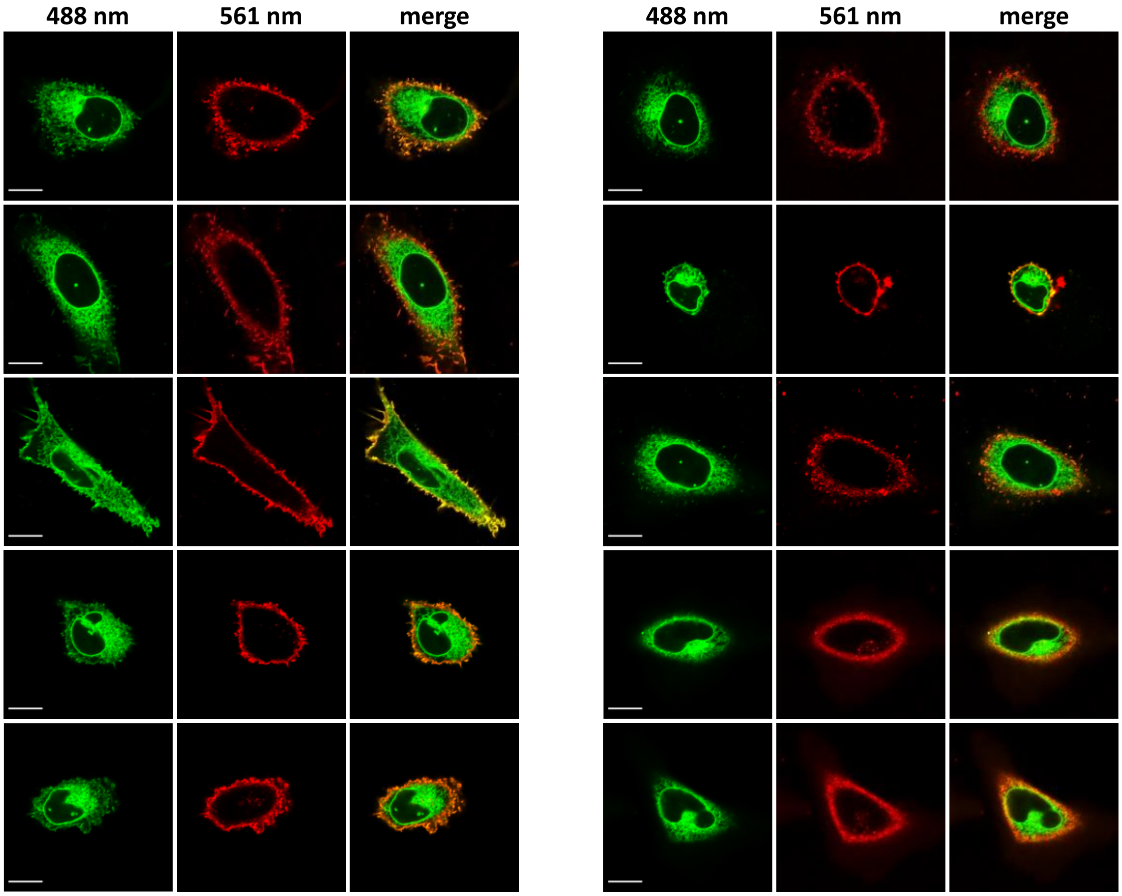

Supplementary Fig. 2. Expression of wildtype NET. Confocal images of mGFP-hNET (488-nm channel) stained with 10 nM AC1-146 (561-nm channel). Scale bars, 10 µm.

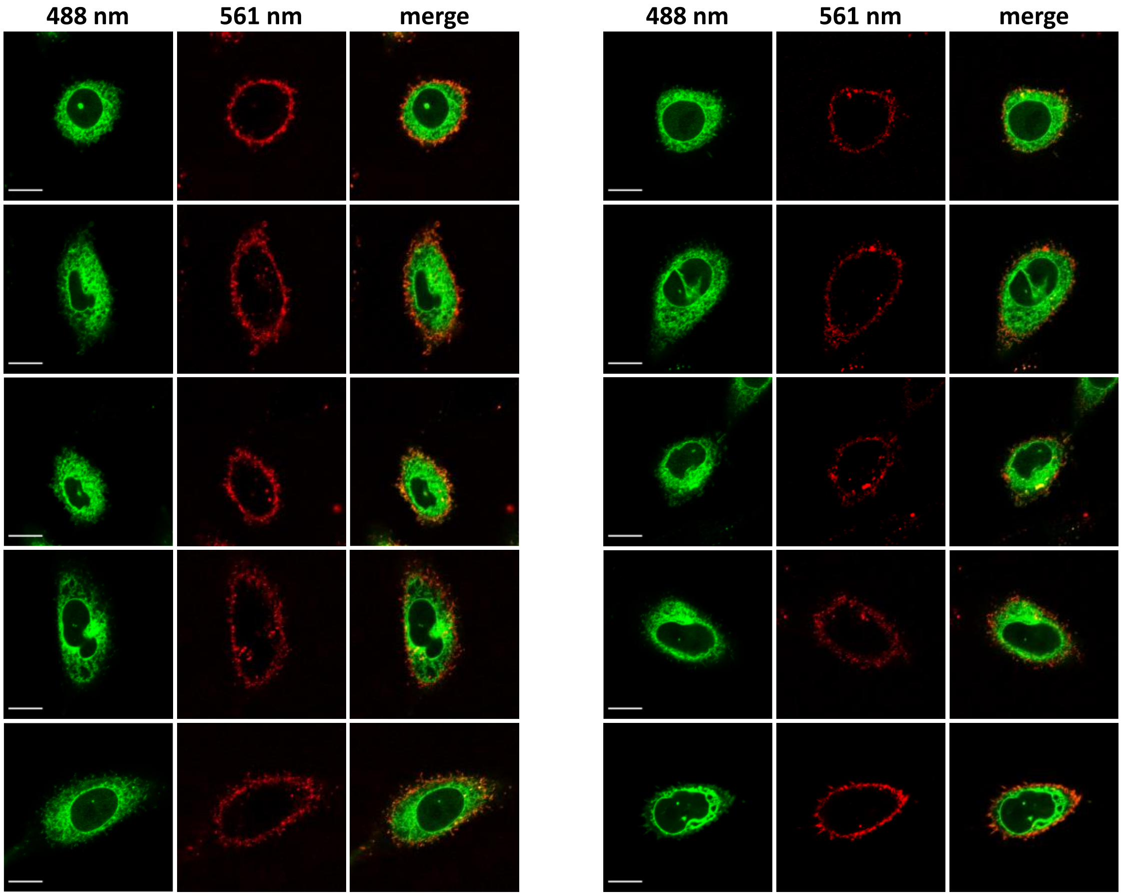

Supplementary Fig. 3. Expression of the RKR/AAA mutant. Confocal images of mGFP-hNET-R121A-K334A-R440A (488-nm channel) stained with 10 nM AC1-146 (561-nm channel). Scale bars, 10 µm.

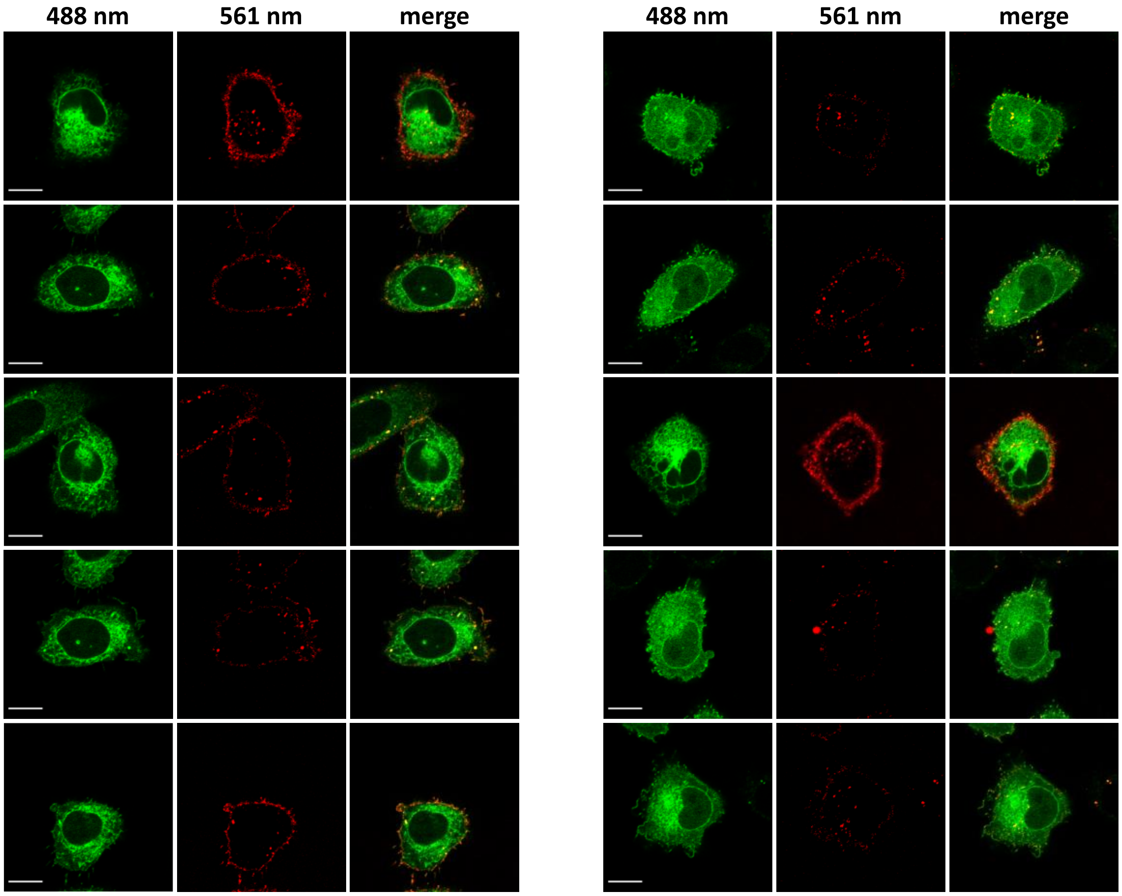

Supplementary Fig. 4. Expression of the A457P mutant. Confocal images of mGFP-hNET-A457P (488-nm channel) stained with 10 nM AC1-146 (561-nm channel). Scale bars, 10 µm.

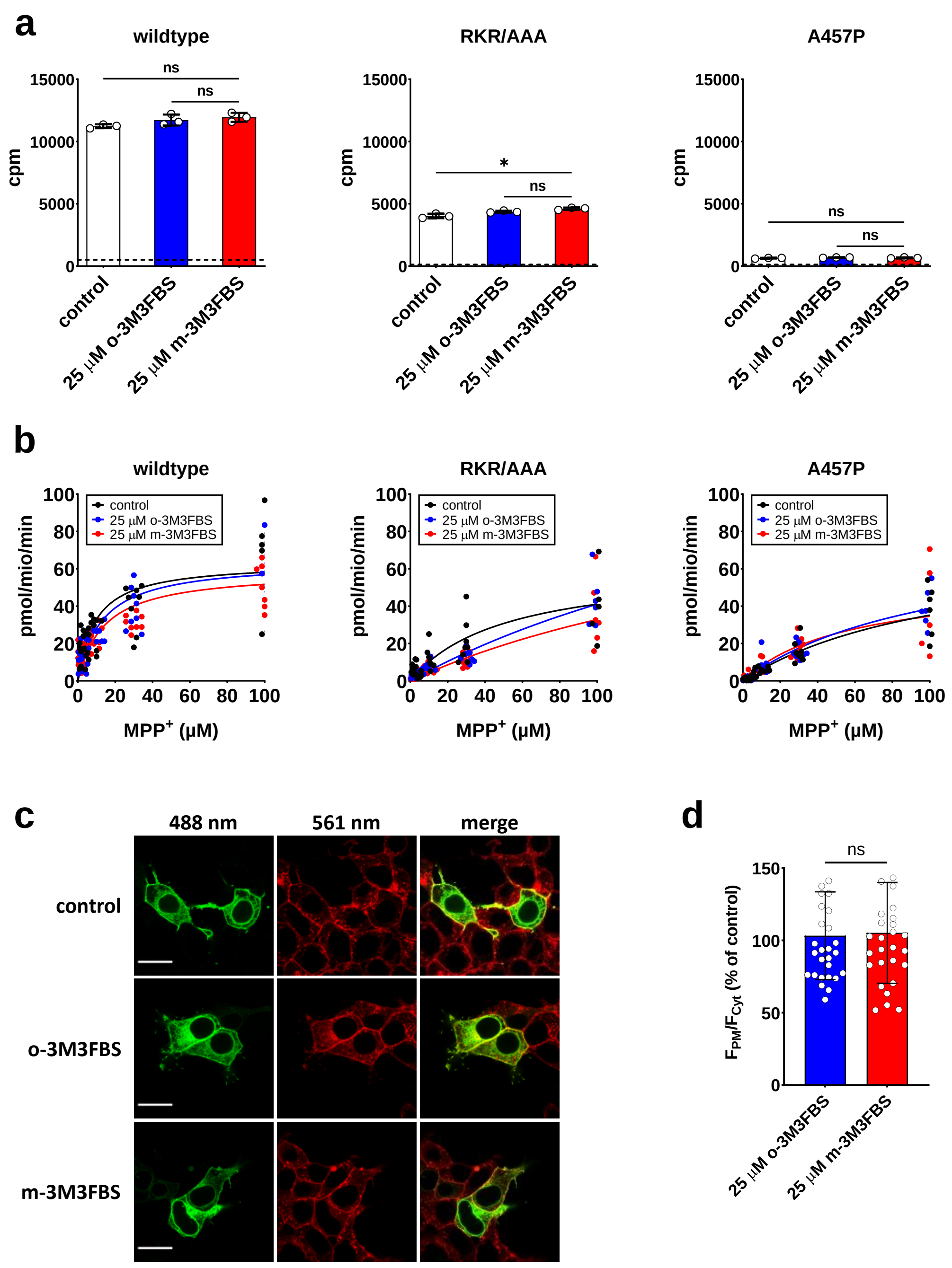

Supplementary Fig. 5. PIP$_2$ depletion does not affect substrate uptake. a) Compared to treatment with the inactive analog o-3M3FBS, PIP$_2$ depletion with m-3M3FBS did not significantly alter substrate uptake of wildtype NET or any of the mutants. For the RKR/AAA mutant, PIP$_2$ depletion resulted in an increased uptake compared to vehicle control. Bars represent means ± SD. b) Substrate saturation of wildtype NET and mutants is not affected by PIP$_2$ depletion. c) Representative confocal images of mGFP-hNET-A457P (488-nm channel) after 13 min treatment with 25 µM m-3M3FBS or o-3M3FBS. The plasma membrane was stained with trypan blue (561-nm channel). Scale bars, 20 µm. d) For mGFP-hNET, PIP$_2$ depletion did not alter the ratio of plasma membrane fluorescence (F_{PM}) to cytosolic fluorescence (F_{Cyt}), indicating equal plasma membrane expression.

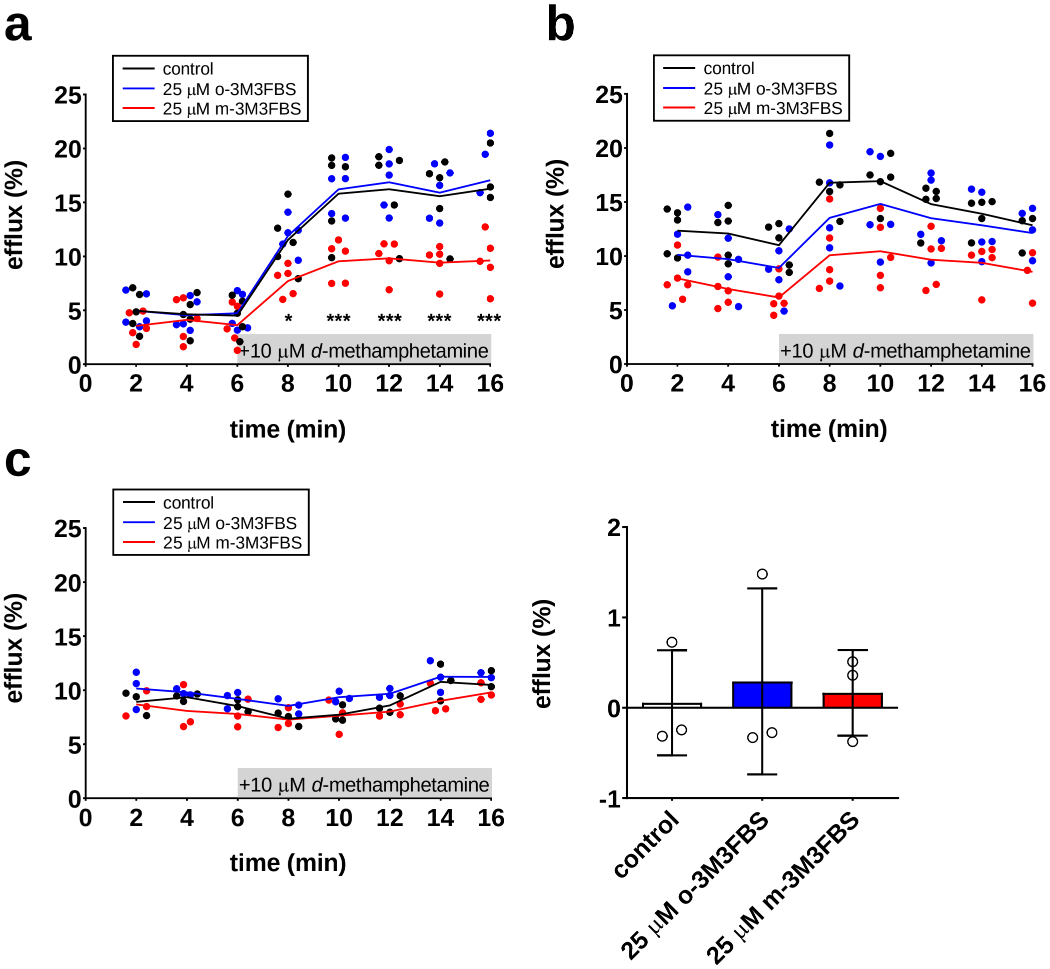

Supplementary Fig. 6. Substrate efflux of the RKR/AAA and A457P mutants is reduced compared to wildtype. a) Substrate-induced efflux curve of wildtype NET after treatment with 25 µM m-3M3FBS or o-3M3FBS. b) Substrate-induced efflux curve of the RKR/AAA mutant after treatment with 25 µM m-3M3FBS or o-3M3FBS. c) The A457P mutant did not release any substrate after treatment with 10 µM *d*-methamphetamine.

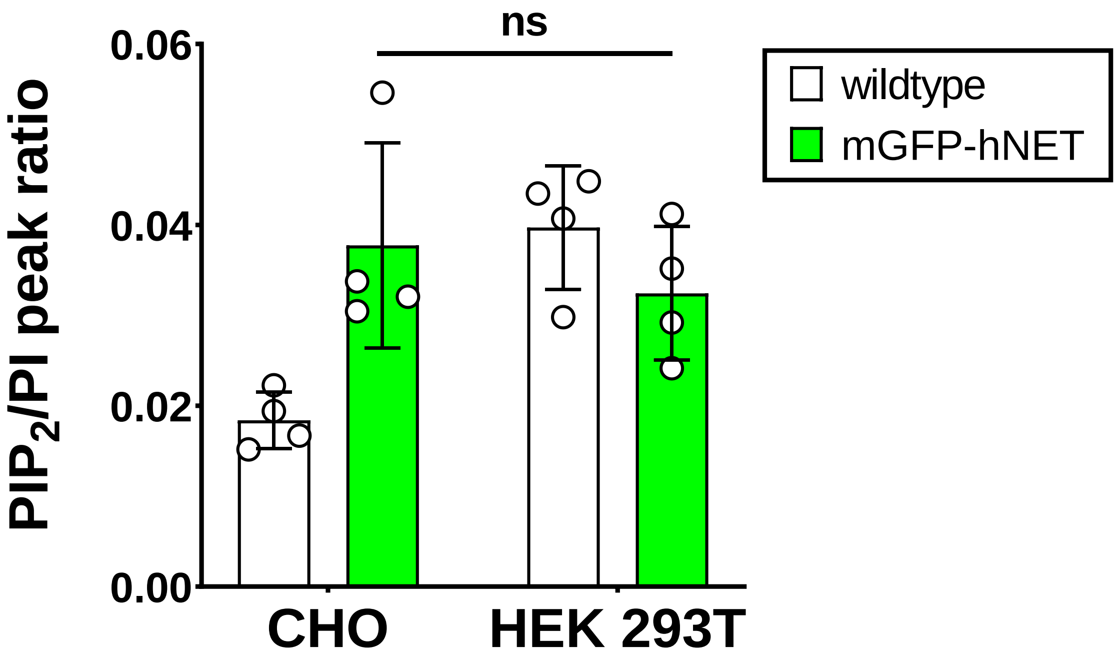

Supplementary Fig. 7. PIP_2 concentrations in CHO and HEK 293T cells transfected with mGFP-hNET are comparable. PIP_2 signals were normalized to total cellular PI. Bars represent means ± SD.

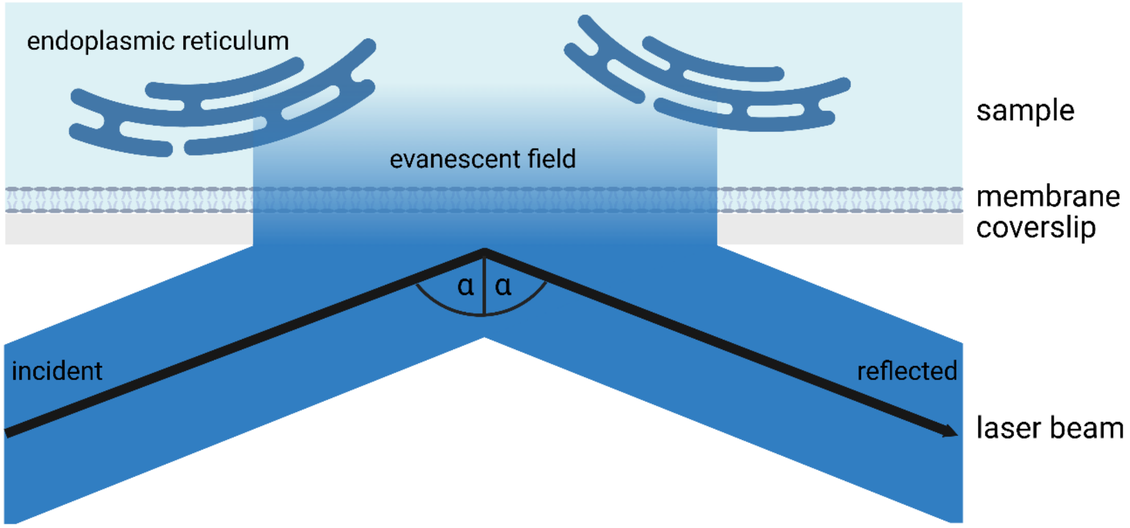

Supplementary Fig. 8. TIRF excitation of the endoplasmic reticulum. In TIRF mode, the evanescent field may penetrate into the sample to a depth of about 100 nm; it may therefore potentially excite fluorophores at the endoplasmic reticulum in addition to those at the cell membrane.

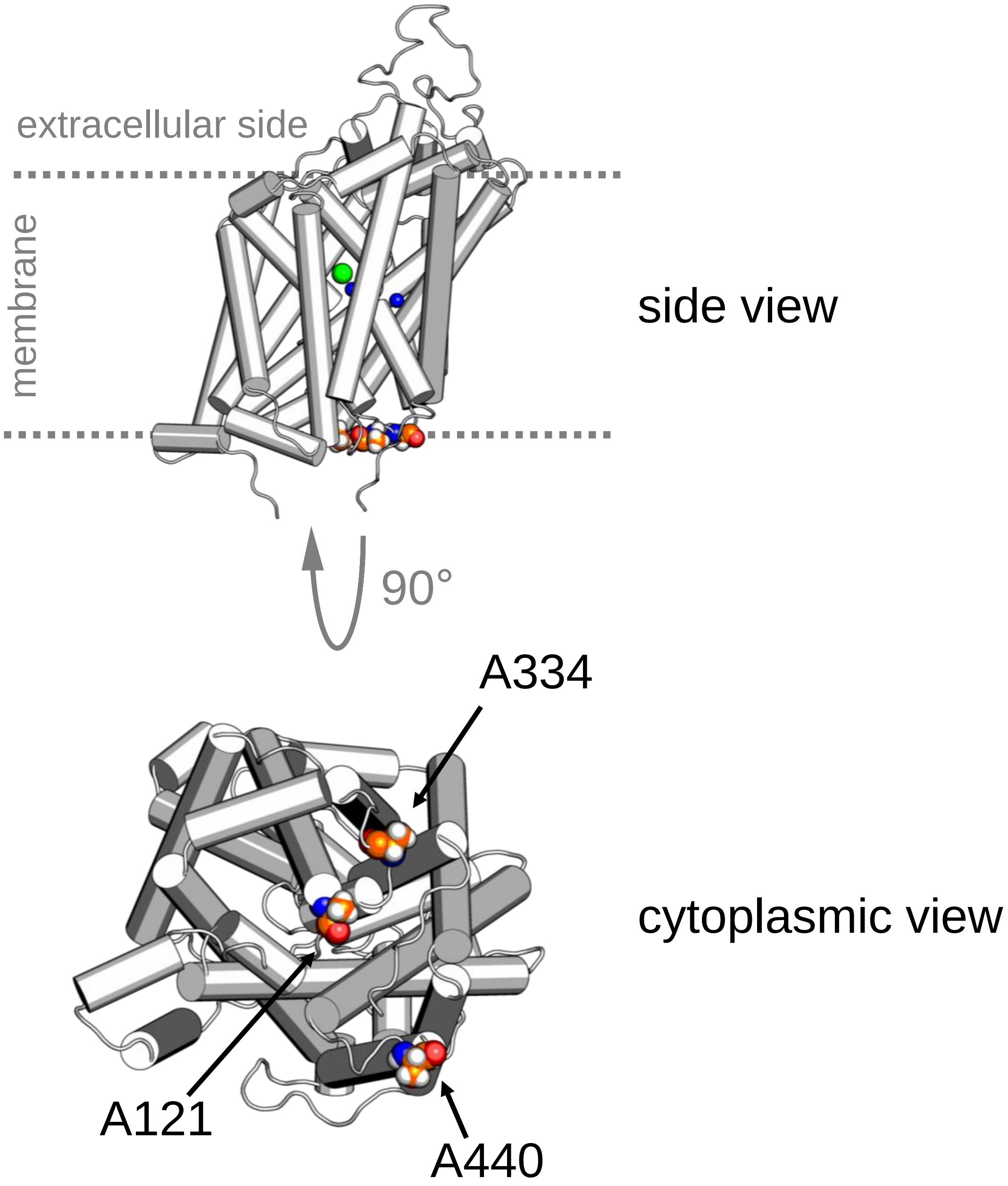

Supplementary Fig. 9. Homology model of the RKR/AAA mutant. The putative PIP_2 binding residues R121, K334, and R440 are mutated to uncharged alanines.

Supplementary Table 1. Substrate efflux in transporter-transfected HEK 293T cells.

		control		o-3M3FBS		m-3M3FBS	
		efflux ± SD (%)	increase *vs.* basal efflux (%)	efflux ± SD (%)	increase *vs.* basal efflux (%)	efflux ± SD (%)	increase *vs.* basal efflux (%)
**wildtype**							
	basal	4.7 ± 1.6	0	4.7 ± 1.5	0	3.8 ± 1.7	0
	8 min	11.5 ± 2.9	145	11.9 ± 1.7	151	7.7 ± 1.4	105
	10 min	15.8 ± 4.0	237	16.2 ± 2.4	242	9.5 ± 1.9	153
	12 min	16.2 ± 4.0	245	16.9 ± 2.6	256	9.8 ± 1.8	161
	14 min	15.6 ± 3.6	232	15.9 ± 2.5	235	9.4 ± 1.7	150
	16 min	16.3 ± 4.5	247	17.1 ± 3.4	260	9.6 ± 2.4	155
**RKR/AAA**							
	basal	11.8 ± 2.1	0	10.1 ± 9.6	0	7.0 ± 1.8	0
	8 min	16.8 ± 2.9	42	13.5 ± 5.1	41	10.1 ± 3.4	44
	10 min	16.9 ± 2.2	43	14.8 ± 4.4	55	10.4 ± 3.1	49
	12 min	14.8 ± 2.1	25	13.5 ± 3.7	41	9.7 ± 2.5	38
	14 min	13.9 ± 1.6	18	12.8 ± 3.0	34	9.4 ± 1.9	34
	16 min	12.8 ± 1.6	9	12.1 ± 2.2	27	8.5 ± 1.8	22
**A457P**							
	basal	8.9 ± 0.7	0	9.7 ± 1.1	0	8.2 ± 1.4	0
	8 min	7.4 ± 0.6	-18	8.5 ± 0.7	-12	7.3 ± 1.0	-11
	10 min	7.7 ± 0.8	-13	9.4 ± 0.5	-4	7.6 ± 1.6	-7
	12 min	8.6 ± 0.8	-4	9.7 ± 0.4	0	8.0 ± 0.6	-2
	14 min	10.8 ± 1.7	21	11.2 ± 1.5	16	9.0 ± 1.4	10
	16 min	10.5 ± 1.3	17	11.2 ± 0.4	16	9.8 ± 0.8	20

Cells were preloaded with MPP^+ and treated with 10 µM *d*-methamphetamine at t = 6 min.
