# Supplementary material for: Phosphatidylinositol 4,5-bisphosphate (PIP2) facilitates norepinephrine transporter dimerization and modulates substrate efflux
Source: Commun Biol. 2022 Nov 17;5:1259. doi: 10.1038/s42003-022-04210-1 (PMC9672106; doi:10.1038/s42003-022-04210-1)
Supplement: Supplementary file 2 — Reporting Summary [file 42003_2022_4210_MOESM2_ESM.pdf]

nature portfolio

Corresponding author(s): Prof. Harald H. Sitte, Prof. Gerhard J. Schütz

Last updated by author(s): May 11, 2022

Reporting Summary

Nature Portfolio wishes to improve the reproducibility of the work that we publish. This form provides structure for consistency and transparency in reporting. For further information on Nature Portfolio policies, see our Editorial Policies and the Editorial Policy Checklist.

Statistics

For all statistical analyses, confirm that the following items are present in the figure legend, table legend, main text, or Methods section.

n/a	Confirmed	
☐	☒	The exact sample size (*n*) for each experimental group/condition, given as a discrete number and unit of measurement
☐	☒	A statement on whether measurements were taken from distinct samples or whether the same sample was measured repeatedly
☐	☒	The statistical test(s) used AND whether they are one- or two-sided<br>*Only common tests should be described solely by name; describe more complex techniques in the Methods section.*
☐	☒	A description of all covariates tested
☐	☒	A description of any assumptions or corrections, such as tests of normality and adjustment for multiple comparisons
☐	☒	A full description of the statistical parameters including central tendency (e.g. means) or other basic estimates (e.g. regression coefficient) AND variation (e.g. standard deviation) or associated estimates of uncertainty (e.g. confidence intervals)
☒	☐	For null hypothesis testing, the test statistic (e.g. *F*, *t*, *r*) with confidence intervals, effect sizes, degrees of freedom and *P* value noted<br>*Give P values as exact values whenever suitable.*
☒	☐	For Bayesian analysis, information on the choice of priors and Markov chain Monte Carlo settings
☒	☐	For hierarchical and complex designs, identification of the appropriate level for tests and full reporting of outcomes
☐	☒	Estimates of effect sizes (e.g. Cohen's *d*, Pearson's *r*), indicating how they were calculated

Our web collection on statistics for biologists contains articles on many of the points above.

Software and code

Policy information about availability of computer code

Data collection	Single molecule data were assessed with custom written software in LabVIEW (National Instruments, Austin, TX, USA). Homology modelling was performed with MODELLER 9.20. Confocal images were recorded using the Nikon NIS-Elements platform (Nikon, Minato City, Tokyo, Japan).
Data analysis	TOCCSL images were analyzed in MATLAB (Mathworks, Portola Valley, CA, USA) using an in-house algorithm (https://github.com/schuetzgroup/TOCCSL_analysis). Data were fitted and statistically analyzed with GraphPad Prism (version 9.3.1, San Diego, CA, USA).

For manuscripts utilizing custom algorithms or software that are central to the research but not yet described in published literature, software must be made available to editors and reviewers. We strongly encourage code deposition in a community repository (e.g. GitHub). See the Nature Portfolio guidelines for submitting code & software for further information.

Data

Policy information about availability of data

All manuscripts must include a data availability statement. This statement should provide the following information, where applicable:

- Accession codes, unique identifiers, or web links for publicly available datasets
- A description of any restrictions on data availability
- For clinical datasets or third party data, please ensure that the statement adheres to our policy

Data supporting the findings of this study are available within the article and supplementary information and at www.zenodo.org upon reasonable request (DOI will be be inserted after acceptance).

Field-specific reporting

Please select the one below that is the best fit for your research. If you are not sure, read the appropriate sections before making your selection.

☒ Life sciences ☐ Behavioural & social sciences ☐ Ecological, evolutionary & environmental sciences

For a reference copy of the document with all sections, see nature.com/documents/nr-reporting-summary-flat.pdf

Life sciences study design

All studies must disclose on these points even when the disclosure is negative.

Sample size	The amount of cells recorded per day was determined based on preliminary experiments assessing the cell viability in imaging buffer over time. Per recorded cell, a few hundred single molecule signals were typically measured. This resulted in thousands of data point per condition, which was determined to be sufficient.
Data exclusions	Exclusion criteria were pre-established. Specifically, single molecule data were excluded if the recording of the cell was of insufficient quality for analysis (i.e., poor TIRF illumination, focus drift over time, or high background noise).
Replication	Experiments were conducted on five different days using different cell passages. The standard deviation of all experiments was calculated to assure the replicability of the experiments.
Randomization	The order of the different experimental conditions was changed with each experiment to exclude any potential bias caused by measurement order.
Blinding	For radiotracer experiments, the experimenter was unaware of the identity of the tested transporter construct. Sample preparation and treatment conditions for single molecule experiments could not be blinded. However, raw data of all conditions were simultaneously analyzed, thereby excluding potential influence of the condition on the analysis.

Reporting for specific materials, systems and methods

We require information from authors about some types of materials, experimental systems and methods used in many studies. Here, indicate whether each material, system or method listed is relevant to your study. If you are not sure if a list item applies to your research, read the appropriate section before selecting a response.

Materials & experimental systems

n/a	Involved in the study	
☒	☐	Antibodies
☐	☒	Eukaryotic cell lines
☒	☐	Palaeontology and archaeology
☒	☐	Animals and other organisms
☒	☐	Human research participants
☒	☐	Clinical data
☒	☐	Dual use research of concern

Methods

n/a	Involved in the study	
☒	☐	ChIP-seq
☒	☐	Flow cytometry
☒	☐	MRI-based neuroimaging

Eukaryotic cell lines

Policy information about cell lines

Cell line source(s)	CHO cells: cat#: 85050302, Sigma-Aldrich, Vienna, Austria; HEK293T cells: cat#: HCL4517, Thermo Fisher Scientific, Vienna, Austria.
Authentication	None of the cell lines used were authenticated.
Mycoplasma contamination	Cell lines were regularly tested negative for mycoplasma contamination using MycoAlert Mycoplasma Detection Kit and MycoAlert Assay Control Set (cat#: LT07-418 and LT07-518, respectively, Lonza, Basel, Switzerland).
Commonly misidentified lines (See ICLAC register)	None of the used cell lines are listed in the ICLAC register.
